# COVID-19 deaths in children and young people in England, March 2020 to December 2021: An active prospective national surveillance study

**DOI:** 10.1371/journal.pmed.1004118

**Published:** 2022-11-08

**Authors:** Marta Bertran, Zahin Amin-Chowdhury, Hannah G. Davies, Hester Allen, Tom Clare, Chloe Davison, Mary Sinnathamby, Giulia Seghezzo, Meaghan Kall, Hannah Williams, Nick Gent, Mary E. Ramsay, Shamez N. Ladhani, Godwin Oligbu

**Affiliations:** 1 Immunisation and Vaccine Preventable Diseases Division, UK Health Security Agency, London, United Kingdom; 2 Paediatric Infectious Diseases Research Group, St George’s University of London, London, United Kingdom; 3 COVID-19 National Epidemiology Cell, UK Health Security Agency, London, United Kingdom; 4 Joint Modelling Team (JMT), UK Health Security Agency, London, United Kingdom; 5 Emergency Preparedness, Response and Resilience, UK Health Security Agency, Porton Down, United Kingdom; PLOS Medicine Editorial Board, UNITED STATES

## Abstract

**Background:**

Coronavirus Disease 2019 (COVID-19) deaths are rare in children and young people (CYP). The high rates of asymptomatic and mild infections complicate assessment of cause of death in CYP. We assessed the cause of death in all CYP with a positive Severe Acute Respiratory Syndrome Coronavirus 2 (SARS-CoV-2) test since the start of the pandemic in England.

**Methods and findings:**

CYP aged <20 years who died within 100 days of laboratory-confirmed SARS-CoV-2 infection between 01 March 2020 and 31 December 2021 in England were followed up in detail, using national databases, surveillance questionnaires, post-mortem reports, and clinician interviews.

There were 185 deaths during the 22-month follow-up and 81 (43.8%) were due to COVID-19. Compared to non-COVID-19 deaths in CYP with a positive SARS-CoV-2 test, death due to COVID-19 was independently associated with older age (aOR 1.06 95% confidence interval (CI) 1.01 to 1.11, *p* = 0.02) and underlying comorbidities (aOR 2.52 95% CI 1.27 to 5.01, *p* = 0.008), after adjusting for age, sex, ethnicity group, and underlying conditions, with a shorter interval between SARS-CoV-2 testing and death. Half the COVID-19 deaths (41/81, 50.6%) occurred within 7 days of confirmation of SARS-CoV-2 infection and 91% (74/81) within 30 days. Of the COVID-19 deaths, 61 (75.3%) had an underlying condition, especially severe neurodisability (*n* = 27) and immunocompromising conditions (*n* = 12). Over the 22-month surveillance period, SARS-CoV-2 was responsible for 1.2% (81/6,790) of all deaths in CYP aged <20 years, with an infection fatality rate of 0.70/100,000 SARS-CoV-2 infections in this age group estimated through real-time, nowcasting modelling, and a mortality rate of 0.61/100,000. Limitations include possible under-ascertainment of deaths in CYP who were not tested for SARS-CoV-2 and lack of direct access to clinical data for hospitalised CYP.

**Conclusions:**

COVID-19 deaths remain extremely rare in CYP, with most fatalities occurring within 30 days of infection and in children with specific underlying conditions.

## Abstract 2

Using national surveillance data, Marta Bertran and colleagues aim to understand the characteristics of children and young people who died of COVID-19 in England between March 2020 and December 2021.

## Author summary

**Why was this study done?:**

- Children and young people (CYP) usually remain asymptomatic or develop mild Coronavirus Disease 2019 (COVID-19) disease compared to adults.
- Death due to COVID-19 in CYP is rare, but there are very few population-based studies on infection fatality rates in CYP, especially with the emergence of new Severe Acute Respiratory Syndrome Coronavirus 2 (SARS-CoV-2) variants.
- The study aimed to understand the characteristics of CYP who died of COVID-19 in England between March 2020 and December 2021.

**What did the researchers do and find?:**

- We linked multiple national datasets to identify all CYP aged <20 years who died within 100 days of a positive SARS-CoV-2 test in England.
- Detailed follow-up identified 185 fatalities over the 22-month surveillance period, and 81 (43.8%) were attributed to COVID-19.
- Approximately 75% had significant underlying health conditions, mainly severe neurodisability, and immunocompromising conditions.
- SARS-CoV-2 was responsible for 1.2% (81/6,790) of all deaths in <20 year olds, with an infection fatality rate of 0.70/100,000 SARS-CoV-2 infections.

**What do these findings mean?:**

- Death due to COVID-19 is very low in CYP, even with the emergence of new SARS-CoV-2 variants.
- Most COVID-19 deaths occurred in CYP with underlying health conditions.

## Introduction

On 11 March 2020, the World Health Organization declared a global pandemic caused by Severe Acute Respiratory Syndrome Coronavirus 2 (SARS-CoV-2), the virus responsible for Coronavirus Disease 2019 (COVID-19) [1]. Two years on, there have been more than 500 million confirmed cases and 6 million deaths reported worldwide [2]. In England alone, more than 18 million cases have been confirmed by 4 April 2022, with almost 158,000 deaths with COVID-19 recorded on the death certificate [3]. Although studies at the start of the pandemic suggested that children may be less susceptible than adults [4], wider testing and surveillance indicate that children are as likely to be infected with SARS-CoV-2 as adults [5]. In England, national testing data showed that, by 01 May 2022, 1 in 4 SARS-CoV-2-confirmed cases (24.3%) were in children and young people (CYP) aged <20 years [3,6], who accounted for 23.6% of the English population in 2020 [7]. In contrast to adults, and particularly older adults who have a high risk of severe and fatal COVID-19, most CYP have asymptomatic infection or a mild, transient, and often non-specific illness when exposed to SARS-CoV-2 [8–10]. Severe COVID-19 requiring hospitalisation is uncommon in CYP, and intensive care admission even less so, occurring mainly in CYP with serious underlying medical conditions [10–12]. Consequently, death due to COVID-19 is extremely rare in CYP, even among those with underlying conditions [13,14]. During the first 12 months of the pandemic in England, of 12,023,568 CYP aged 18 years in England, there were 3,105 deaths, including 61 who were positive for SARS-CoV-2 [15]. Of these, 25 were due to SARS-CoV-2 infection (2 deaths per million), including 22 directly attributable to COVID-19 and 3 to paediatric inflammatory multisystem syndrome (PIMS-TS, also known as multisystem inflammatory syndrome in children, MIS-C) [15]. Of these 25 deaths, 19 had significant underlying conditions, including 15 with life-limiting conditions [15].

Understanding the risk of fatal COVID-19 is important for parents, clinicians, and policy makers, especially in the context of emerging variants and increasing availability of effective preventatives, including vaccines and immunoglobulins, and treatments for COVID-19. In England, death within 28 days of SARS-CoV-2 infection are routinely reported but cause of death cannot be inferred [16,17], especially for CYP, who have high rates of incidental infections on a background of complex underlying conditions [15]. Consequently, children with a positive SARS-CoV-2 test who die of other causes and those who succumb to their underlying conditions will be included in those figures. More recently, death registrations with mention of SARS-CoV-2/COVID-19 are routinely reported by the UK Office for National Statistics (ONS)[18] but, as we and others have previously reported, these too are limited by the information available on the death certificate, making it difficult to assess the contribution of SARS-CoV-2 infection to death [19,20]. During the first 12 months of the pandemic, for example, only 40% of deaths in CYP with confirmed infection in England were due to COVID-19 [15]. The problem of incidental infections is likely to be exacerbated with free and widely available testing for SARS-CoV-2, including home testing with lateral-flow device (LFD) testing since March 2021 [21]. In the United States, the Center for Disease Prevention and Control (CDC) real-time COVID-19 tracker removed 416 childhood deaths from its national statistics after it became apparent that previously reported figures included children with incidental SARS-CoV-2 infection [22].

Since the last detailed analysis of childhood COVID-19 deaths, which included the first pandemic wave in March 2020 and the Alpha variant wave since November 2021 [15], England has experienced 2 further waves due to the Delta variant since May 2021 and Omicron since November 2021. Although the Delta variant was associated with a 2-fold higher risk of severe COVID-19 in adults, we recently reported very low rates of hospitalisations, with no deaths, in nearly 150,000 children infected with the Alpha or Delta variant [23]. Currently, there are limited data on childhood COVID-19 deaths, especially with the more recent SARS-CoV-2 variants.

In England, the UK Health Security Agency (UKHSA, formerly Public Health England) has been conducting COVID-19 surveillance in CYP since the start of the pandemic [24,25], including detailed follow-up of all fatalities in CYP with confirmed SARS-CoV-2 infection to ascertain the cause of death. Here, we report our surveillance of all deaths in CYP within 100 days of confirmed SARS-CoV-2 infection from the start of the pandemic until December 2021.

## Methods

As part of the public health response to the global COVID-19 pandemic, the UKHSA routinely receives daily reports of all laboratory-confirmed SARS-CoV-2 infections (cases) in England through the Second-Generation Surveillance System (SGSS), an electronic database used by National Health Service (NHS) laboratories to report significant infections to UKHSA [26]. Confirmed SARS-CoV-2 infections in healthcare settings (pillar 1) and in the community (pillar 2) between 01 March 2020 and 31 December 2021 (22 months) in CYP aged <20 years were extracted from SGSS and linked to electronic registrations records provided by the ONS to UKHSA for public health surveillance and to the Personal Demographic Service (PDS), a real-time electronic database of patients registered with the NHS, with information on demographics, name, address of their general practitioner, current status (alive/dead), and date of death [27]. Records were linked by using combinations of unique individual NHS number, first name, last name, date of birth, sex, and postcode. Cases were also linked using the same identifiers to Hospital Episodes Statistics (HES), an electronic administrative database containing details of all admissions, outpatient appointments, and emergency department attendances at NHS hospitals, to obtain further details on medical history and cause of death [28].

The general practitioners of children who died were requested to complete a short surveillance questionnaire—online using the SnapSurvey software [Snap Surveys, Portsmouth, New Hampshire] or on paper—requesting information about the reason for SARS-CoV-2 testing, symptoms, underlying conditions, hospitalisations, intensive care admission, and cause of death. We also requested general practitioners to provide copies of hospital discharge summaries, death certificates, and post-mortem reports if performed. Unreturned and incomplete questionnaires were followed up by phone and email. Where general practitioners were unable to provide sufficient information to ascertain the cause of death, we contacted hospital clinicians, the local designated doctor for child death, and, where post-mortems were performed, pathologists and coroners for more information, as needed. Data for individual cases were reviewed independently by at least 2 authors and any conflicts were resolved through discussion with other authors. A COVID-19 death was defined as any fatality in a CYP with a positive SARS-CoV-2 test who died within 100 days of the test where the virus contributed to the death. Non-COVID-19 deaths included deaths in a CYP with a positive SARS-CoV-2 test who died within 100 days that were not related to SARS-CoV-2 infection and had a clear alternative cause, as well as deaths in CYP who survived their infection but died later due to a different cause, including their underlying medical condition. Where there was insufficient information to ascertain whether the SARS-CoV-2 infection contributed to the death (such as lack of death certificate, port-mortem, or coroner’s report) [29], the authors discussed the case with the clinician responsible for the CYP’s care over the telephone to decide the most reasonable classification. Sudden deaths and deaths with plausible clinic with no other identified or confirmed cause of death were included as COVID-19 deaths (*n* < 5). Stillbirths with a positive SARS-CoV-2 test at post-mortem were excluded.

### Statistical analyses

Responses for the surveillance questionnaire completed online were downloaded and imported into a surveillance database (Microsoft Access), which contained responses from paper questionnaires that were manually entered. Data were cleaned, de-duplicated, and imported into Stata 15.1 (StataCorp LLC, College Station, Texas, United States of America) for analysis. We described demographics (age, sex, ethnicity) using proportions for categorical data and medians with interquartile ranges (IQRs) for continuous variables. Age at death was grouped into <1, 1 to 4, 5 to 11 (primary school-aged), 12 to 15 (secondary school-aged), and 16 to 19 (higher education-aged) years. We categorised CYP by educational years because they were offered COVID-19 vaccination at different time points during the surveillance period: healthy CYP 18+ from June 2021 [30], CYP aged 12 to 15 years with severe neurodisabilities and immunosuppression from July 2021 [31], 16 to 17 years olds from August 2021 [32], and to 12 to 15 years olds from September 2021 [33]. Children under 12 were not eligible for vaccination during this surveillance period [34]. We classified the predominant underlying condition hierarchically into: immunocompromising, severe neurodisability, other non-immunocompromising conditions, and none. We did not sub-classify non-immunocompromising conditions because of small numbers. The COVID-19 pandemic wave periods were classified according to the predominating variant as follows: wild-type (01/03/2020 to 30/11/2020), Alpha (01/12/2020 to 30/05/2020), and Delta (01/06/2020 to 31/12/2021). We also sought to determine whether the children died at home or were hospitalised at the time of death, and, if so, required paediatric intensive care admission. Monthly SARS-CoV-2 infection rates were calculated using the earliest specimen date for confirmed SARS-CoV-2 infections reported through SGSS stratified by age group. Mortality rates were calculated using ONS mid-year population estimates for 2020 [7], deaths due to all causes were estimated from the ONS figures for England and Wales [18], assuming 93% of the deaths occurred in England, based on the previous 5-year distribution. Infection fatality rates (IFRs) were calculated using the number of COVID-19 deaths as numerators with confirmed and modelled estimates of SARS-CoV-2 infections as denominators [35,36].

To assess factors associated with a COVID-19 death, a multivariable logistic regression model was fitted for CYP who died within 100 days of a positive SARS-CoV-2 test with COVID-19/non-COVID-19 death as the dependent variable and age as a continuous variable, sex, ethnicity group, and underlying conditions as independent variables. There were no missing data in the model. We assessed fatalities by interval between date of positive SARS-CoV-2 test and date of death for both COVID-19 and non-COVID-19 deaths using the Kaplan–Meier method. A log-rank test was performed to assess for equality of survival functions between COVID-19 and non-COVID-19 deaths. A *p*-value of <5% was considered to be statistically significant. Our analysis did not include deaths among cases who tested positive after 30 December 2021 and, as such, excludes deaths during the Omicron wave in England. This study is reported according to the Strengthening the Reporting of Observational Studies in Epidemiology (STROBE) guideline (S1 Checklist).

### Ethics approval

UKHSA has legal permission to process confidential information for national surveillance of communicable diseases without individual patient consent (Regulation 3 of Health Service Regulations 2002 [37]) and, as such, ethics committee approval is not required.

## Results

**Fig 1:**
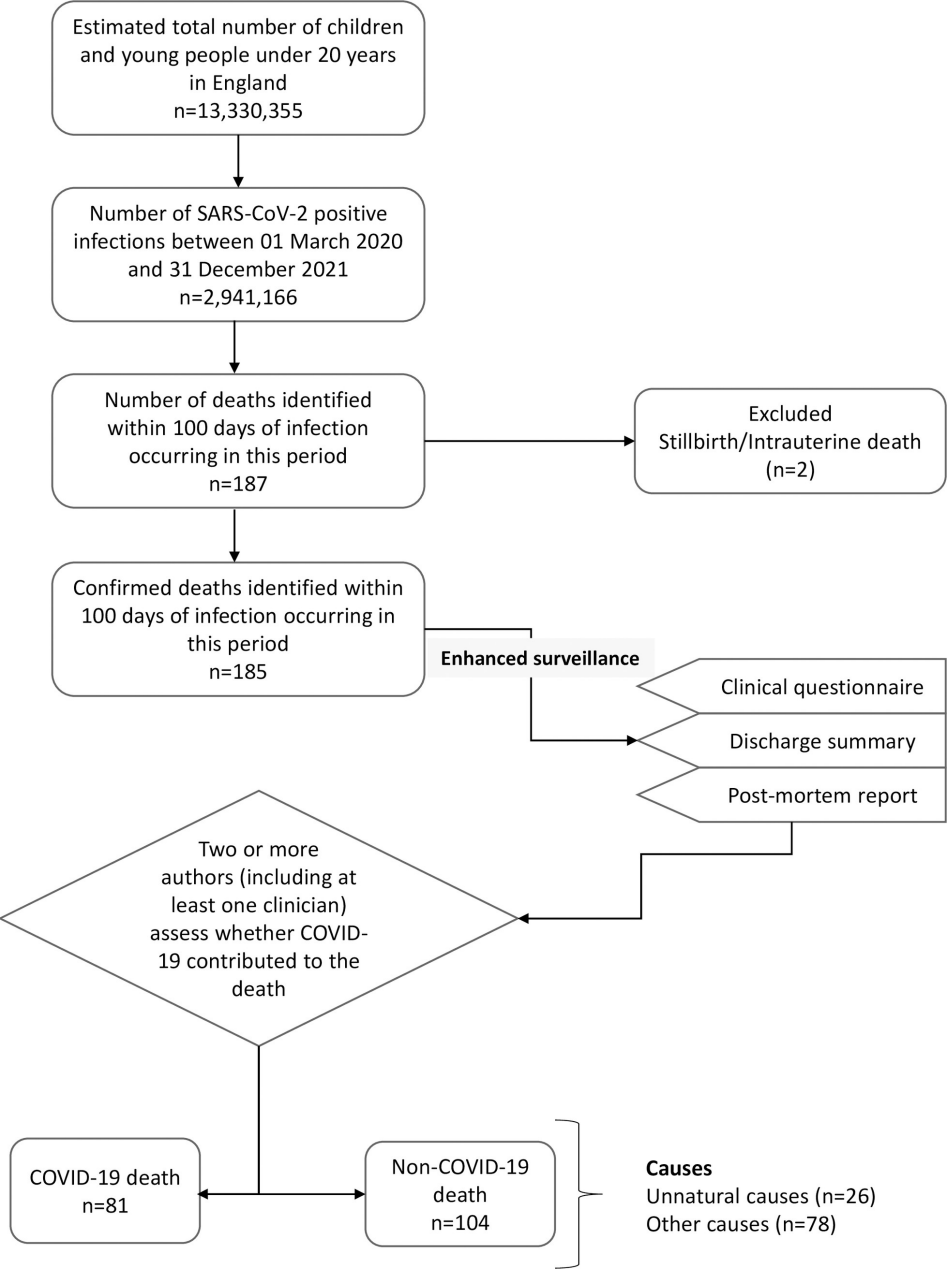
Flow chart of CYP under 20 years with COVID-19 who died within 100 days of COVID-19 between 1 March 2020 and 31 December 2021. COVID-19, Coronavirus Disease 2019; CYP, children and young people; SARS-CoV-2, Severe Acute Respiratory Syndrome Coronavirus 2.

**Fig 2:**
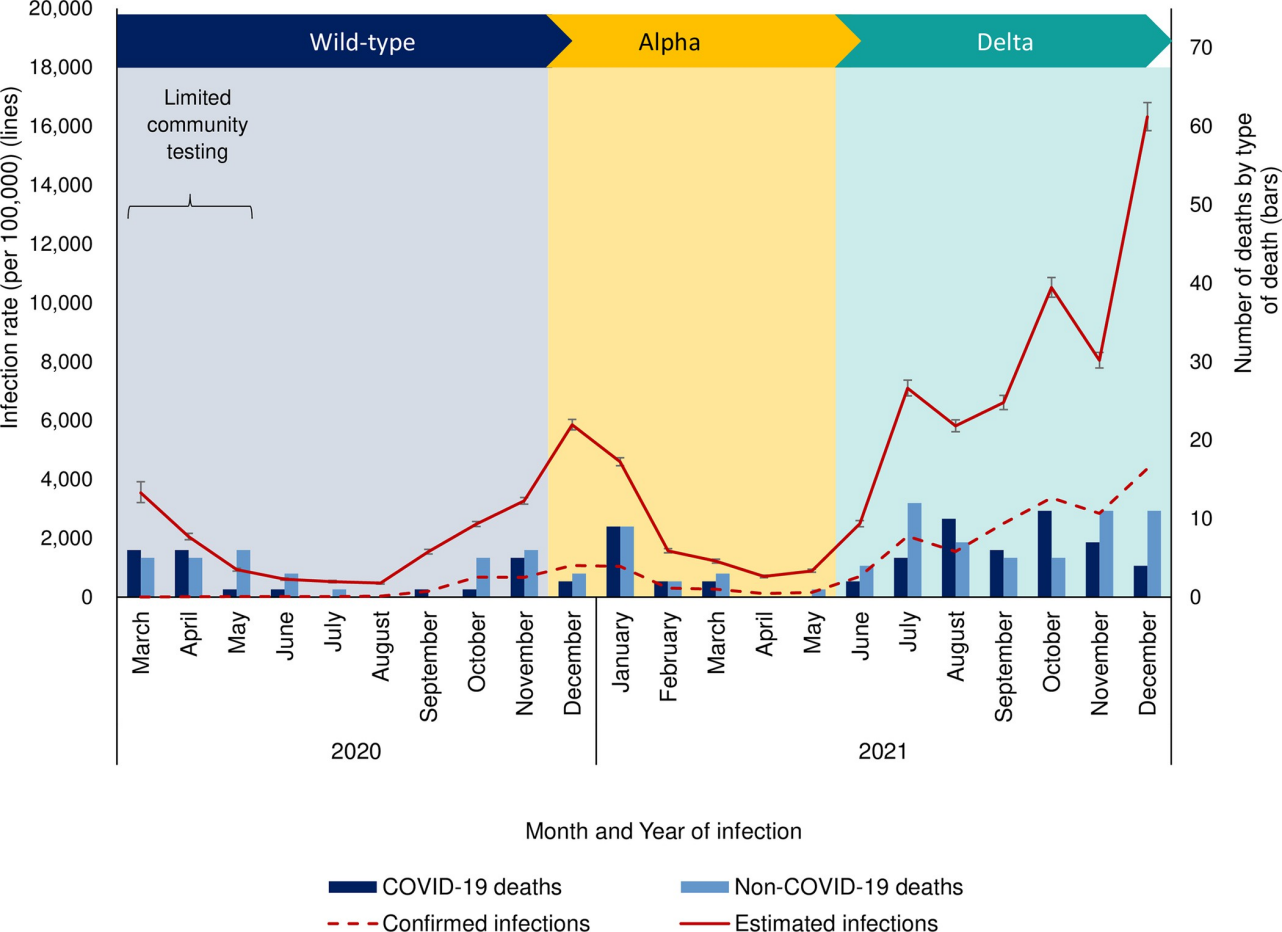
COVID-19 infection rates by age group and number of deaths by cause of death in CYP <20 years (predominant circulating variant shown by coloured chevrons; error bars indicate 95% confidence intervals). (Note non-COVID deaths only include deaths in CYP with a SARS-CoV-2 positive test within 100 days.) COVID-19, Coronavirus Disease 2019; CYP, children and young people; SARS-CoV-2, Severe Acute Respiratory Syndrome Coronavirus 2.

Between 01 March 2020 and 31 December 2021, there were 185 deaths within 100 days of a positive SARS-CoV-2 test in CYP aged <20 years in England, of which 81 (43.8%) were COVID-19 deaths (**Fig 1**). Non-COVID-19 deaths (104/185, 56.2%) were due to unnatural causes (26/104, 25.0%) or due to other causes unrelated to COVID-19 (78/104, 75.0%). The deaths occurred throughout the 22-month surveillance period, mainly during the peaks of the 3 pandemic waves due to wild-type, Alpha, and Delta variants (**Fig 2**).

### COVID-19 deaths vs non-COVID-19 deaths in CYP with a positive SARS-CoV-2 test

Compared to non-COVID-19 deaths in CYP with a positive SARS-CoV-2 test, those who died of COVID-19 were more likely to be older (aOR 1.06 95% CI 1.01 to 1.11, *p* = 0.02) and have underlying comorbidities (aOR 2.52 95% CI 1.27 to 5.01, *p* = 0.008), in particular severe neurodisability, but did not differ significantly by sex or ethnicity in the multivariable logistic regression model (**Table 1**). Among COVID-19 deaths, adolescents were over-represented, with two-thirds of deaths occurring among 16 to 19 (32/81, 39.5%) and 12 to 15 (22/81, 27.2%) year olds (**Table 2**).

**Table 1:** Risk of COVID-19 death in CYP with a SARS-CoV-2 positive test within 100 days by demographics (adjusted and unadjusted odds ratios).

Demographics	Unadjusted odds ratio (95% CI)	*p*-value	Adjusted* odds ratio (95% CI)	*p*-value
**Age**	1.04 (95% CI 0.99–1.08)	0.11	1.06 (95% CI 1.01–1.11)	0.02
**Sex**				
Male	Reference		Reference	
Female	1.64 (95% CI 0.91–2.95)	0.10	1.67 (95% CI 0.87–3.2)	0.12
**Ethnicity**				
White	Reference		Reference	
Asian	2.31 (95% CI 1.16–4.59)	0.02	1.96 (95% CI 0.95–4.02)	0.07
Black	1.48 (95% CI 0.6–3.65)	0.39	1.42 (95% CI 0.55–3.68)	0.47
Mixed	1.31 (95% CI 0.28–6.2)	0.73	1.82 (95% CI 0.35–9.53)	0.48
Other	1.75 (95% CI 0.24–12.96)	0.58	2.07 (95% CI 0.26–16.3)	0.49
**Comorbidities**				
No comorbidities	Reference		Reference	
Comorbid	2.61 (95% CI 1.38–4.93)	0.003	2.52 (95% CI 1.27–5.01)	0.008

* Adjusted for all the variables included in this table.

CI, confidence interval; COVID-19, Coronavirus Disease 2019; CYP, children and young people; SARS-CoV-2, Severe Acute Respiratory Syndrome Coronavirus 2.

**Table 2:** Characteristics of CYP with confirmed SARS-CoV-2 infection who died within 100 days of COVID-19 or other causes (non-COVID-19).

	Cause of death	
	Non-COVID-19 (*n* = 104)	COVID-19 (*n* = 81)
Interval from infection to death (median (IQR*), *days)*	42 (12–73)	6 (2–15)
Age (median (IQR*), *years)*	15 (1–18)	14 (10–17)
Age group		
<1 year	22 (21.2%)	5 (6.2%)
1–4 years	8 (7.7%)	6 (7.4%)
5–11 years	12 (11.5%)	16 (19.8%)
12–15 years	15 (14.4%)	22 (27.2%)
16–19 years	47 (45.2%)	32 (39.5%)
Sex		
Male	64 (61.5%)	40 (49.4%)
Female	40 (38.5%)	41 (50.6%)
Ethnic group		
White	63 (60.6%)	36 (44.4%)
Asian	22 (21.2%)	29 (35.8%)
Black	13 (12.5%)	11 (13.6%)
Mixed	4 (3.8%)	3 (3.7%)
Other	2 (1.9%)	2 (2.5%)
Any comorbidities		
At least 1 comorbidity	56 (53.8%)	61 (75.3%)
None	48 (46.2%)	20 (24.7%)
Comorbidity groups		
Immunocompromising	18 (17.3%)	12 (14.8%)
Severe neurodisability	15 (14.4%)	27 (33.3%)
Other non-immunocompromising	23 (22.1%)	22 (27.2%)
None	48 (46.2%)	20 (24.7%)
Hospitalisation		
Yes	68 (65.4%)	67 (82.7%)
No	36 (34.6%)	14 (17.3%)
Admission to ICU**		
Yes	33 (37.5%)	43 (57.3%)
No	55 (62.5%)	32 (42.7%)
Vaccination status (*n* = 59)***		
At least 1 dose	10 (27.0%)	1 (4.5%)
2 doses	6 (16.2%)	1 (4.5%)

* IQR, interquartile range.

** ICU (intensive care unit) admission status was missing for 16/104 non-COVID-19 and 6/81 COVID-19 deaths.

*** 37 eligible for vaccination among non-COVID-19 death vs. 22 among COVID-19 deaths.

COVID-19, Coronavirus Disease 2019; CYP, children and young people; SARS-CoV-2, Severe Acute Respiratory Syndrome Coronavirus 2.

Only 1/22 (4.5%) CYP eligible for COVID-19 vaccination had received 2 doses at least 14 days prior to their death, compared to 6/37 (16.2%) eligible CYP who died of other causes and 4 others in the latter cohort who had received 1 dose (4/37, 10.8%) (Table 2).

Of the COVID-19 deaths, 61 (75.3%) CYP had an underlying condition as follows: severe neurodisabilities (*n* = 27), immunocompromising conditions (*n* = 12), and other non-immunocompromising conditions (*n* = 22). Among the latter, 11 had a congenital syndrome, including Down syndrome (Trisomy 21) and Edward syndrome (Trisomy 18) (≤5 cases each) and 7 had chronic heart disease. There were 5 COVID-19 deaths in infants (aged <1 year) and 4 had been born prematurely (29 to 36 weeks gestation) (**Table 2**).

**Fig 3:**
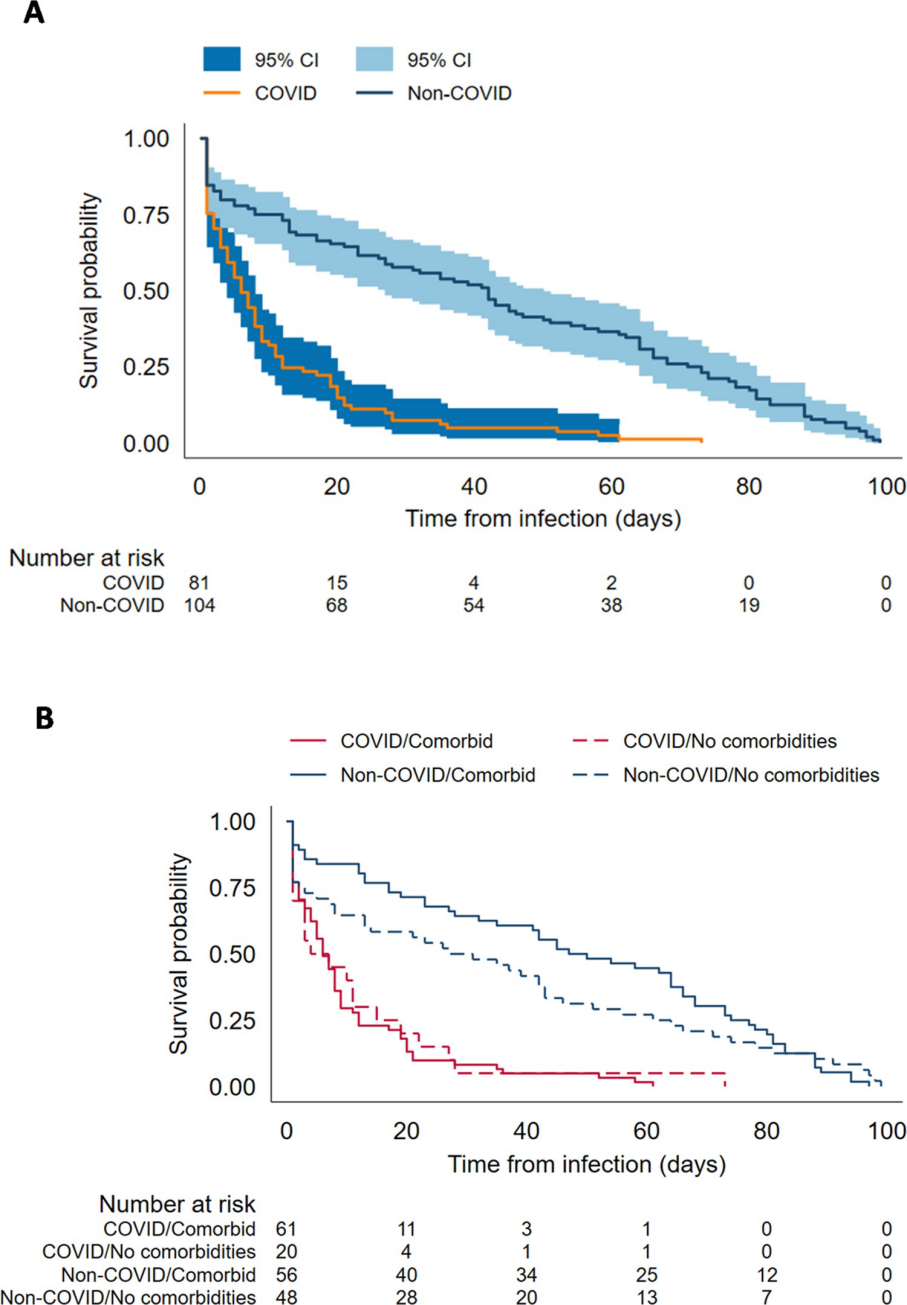
Kaplan–Meier survival estimates of death following COVID-19 by (A) type of death and (B) type of death stratified by comorbidity status. CI, confidence interval; COVID-19, Coronavirus Disease 2019.

Kaplan–Meier analysis identified that COVID-19 deaths occurred more rapidly after confirmation of SARS-CoV-2 infection compared to non-COVID-19 deaths (log-rank test, *p* < 0.001). The median interval for COVID-19 deaths was 6 days (IQR 2–15) compared to 42 days (IQR 12–73) for non-COVID-19 deaths, which occurred more gradually over the 100-day period. This was greater, both in previously healthy CYP and in those with underlying conditions (**Fig 3**). Among the 81 COVID-19 deaths, half (*n* = 41) occurred within 7 days of confirmation of SARS-CoV-2 infection and 91.4% (*n* = 74) within 30 days. When assessed by interval between testing and death, 41/63 (65.1%) deaths within 7 days were due to COVID-19 compared with 74/117 (63.2%) deaths within 30 days and 78/143 (54.5%) deaths within 60 days of confirmation of SARS-CoV-2 infection.

Six COVID-19 deaths occurred more than 30 days after confirmation of SARS-CoV-2 infection. Five had severe underlying conditions and 3 were adolescents (12 to 18 years). They all died after a prolonged hospital stay, either directly because of severe COVID-19 (*n* = 2) or because of a secondary complication of severe COVID-19 (*n* = 4).

### Infection fatality and mortality rates

During the 22-month surveillance period, COVID-19 was responsible for 1.2% (81/6,790) of all deaths in CYP aged <20 years (**Table 3**). Over the same period, there were 2.9 million confirmed and 11.6 million estimated SARS-CoV-2 infections in CYP aged <20 years in England, giving an IFR of 2.8 per 100,000 confirmed SARS-CoV-2 infections and 0.70/100,000 estimated SARS-CoV-2 infections, with an overall mortality rate of 0.61/100,000 CYP. When assessed by variant wave, IFR was lowest during the Delta variant wave but there were more COVID-19 deaths (45 during the Delta wave versus 36 deaths during the wild-type [*n* = 21] and Alpha [*n* = 15] variant waves) and a greater contribution of COVID-19 deaths to total deaths during the Delta wave than the whole pandemic period prior to the Delta variant wave (**Table 3**).

**Table 3:** COVID-19 infection fatality rates and mortality rates, stratified by age group and variant period.

		COVID-19 deaths/total deaths n/N (%)	IFR (using confirmed infections) n/N (per 100,000)	IFR (using estimated infections) n/N (per 100,000)	Mortality rate (per 100,000)
Age group (years)	<1	5/4,142 (0.12%)	5/64,846 (7.7)	5/ 302,064 (1.7)	5/601,913 (0.83)
	1 to 4	6/541 (1.11%)	6/190,011 (3.2)	6/1,767,856 (0.3)	6/2,637,534 (0.23)
	5 to 11	16/517 (3.09%)	16/1,067,742 (1.5)	16/4,964,261 (0.3)	16/4,935,418 (0.32)
	12 to 15	22/494 (4.45%)	22/870,587 (2.5)	22/2,512,558 (0.9)	22/2,677,375 (0.82)
	16 to 19	32/1,096 (2.92%)	32/747,980 (4.3)	32/2,082,668 (1.5)	32/2,478,115 (1.29)
	Total	81/6,790 (1.19%)	81/2,941,166 (2.8)	81/11,629,407 (0.7)	81/13,330,355 (0.61)
Variant period	Wild-type (01 March to 30 November 2020)	21/2,638 (0.80%)	-*	21/2,062,780 (1.0)	-
	Alpha (01 December 2020 to 31 May 2021)	15/1,842 (0.81%)	15/396,851 (3.8)	15/1,980,140 (0.8)	-
	Delta (01 June 2021 to 31 December 2021)	45/2,309 (1.95%)	45/2,321,391 (1.9)	45/7,586,488 (0.6)	-
	Total	81/6,790 (1.19%)	81/2,941,166 (2.8)	81/11,629,407 (0.7)	-

*IFR using confirmed infections has been excluded due to very limited testing in the community, with testing only becoming available gradually from 01 June 2020.

COVID-19, Coronavirus Disease 2019; IFR, Infection fatality rate.

## Discussion

National surveillance identified 185 CYP aged <20 years who died within 100 days of a positive SARS-CoV-2 test between 01 March 2020 and 31 December 2021 in England. Of these, 81 (43.7%) were COVID-19 deaths, contributing to 1.2% of deaths in this age group, with an estimated IFR of 0.7/100,000 and mortality rate of 0.61/100,000 CYP. Most CYP (75.3%) who died of COVID-19 had an underlying medical condition, notably severe neurodisabilities and immunocompromising conditions. Time interval analysis showed that half the COVID-19 deaths occurred within 7 days and 91.4% within a month of testing positive for SARS-CoV-2, with deaths occurring after this period being mainly unrelated to COVID-19.

Two years into the pandemic, there are still limited data on severe and fatal COVID-19 in CYP, mainly because these are uncommon outcomes of infection in CYP. In adults, hospitalisations and deaths have regularly been used to assess the impact of new variants and vaccines on severe disease outcomes [38,39]. This has allowed rapid, large-scale analysis to be undertaken using national datasets. In contrast, the high prevalence of asymptomatic and mild infections in CYP results in high rates of incidental infections in CYP who are hospitalised with other illnesses or die of other causes. Assessing disease severity in vulnerable CYP, who often have severe, multiple, and often life-limiting comorbidities, can therefore be challenging [11,15]. In the ISARIC4C prospective national study of hospitalised cases during January to July 2020, 276/651 (42.4%) CYP aged <19 years had comorbidities and 6/627 (1%) died in hospital, all of whom had profound comorbidity [12]. When comparing population-based studies, our findings are remarkably consistent with the previous publication on childhood deaths during the first pandemic year in England, both in terms of the proportion of deaths attributed to COVID-19 after detailed review of all available data (81/185, 44% versus 25/61, 40.9%), the proportion with underlying conditions (75% versus 76%) with a predominance of neurodisabilities and overrepresentation of older CYP among fatal cases [15]. While the previous study included CYP up to 18 years of age, our surveillance extended to CYP aged <20 years, which accentuated the increasing risk of death with age [15]. In line with that study, too, we also found an overrepresentation of non-White ethnic groups among COVID-19 deaths (56%), which compares to a national prevalence of 15% in England [40]. These differences in ethnic groups may be indicative of wider disparities such as socioeconomic status, cultural differences, or access to healthcare, which are worthy of further study [41]. Our results are also in line with a recent systematic review up to May 2021, which also found higher odds of severe disease and death in CYP aged >10 years and underlying comorbidities, especially neurological or cardiac conditions [42], which is consistent with other published studies [11,15,42,43]. Notably, however, compared to children who died of other causes in England, previous analyses found that underlying health conditions were not overrepresented in children who died of COVID-19 [15,44], or among survivors and non-survivors in children hospitalised for COVID-19 [45].

When assessing all-cause deaths in CYP aged <20 years in England, fewer CYP died during 2020 (*n* = 3,651) or 2021 (*n* = 3,139) compared to an average of 4,140 deaths in the previous 5 years (2015 to 2019) [46], and COVID-19 contributed to only 1.2% of all deaths until the end of 2021, with most deaths occurring in adolescents. Of the confirmed COVID-19 deaths, nearly all fatalities occurred within 30 days of a positive SARS-CoV-2 test, similar to the previous English study [15]. Even within this period, however, only 63% of deaths in CYP with a positive SARS-CoV-2 test were attributable to COVID-19, indicating that 28-day or 30-day infection fatality rates substantially overestimates the risk of death in CYP. At the same time, death certificates were missing for many children at the time of analysis, often because of delays in post-mortem assessments and coroner inquests to ascertain the cause of death in complex cases. Additionally, death certificates contain very limited information to ascertain the contribution of SARS-CoV-2 to the death. Some death registrations documented “SARS-CoV-2” or “COVID-19” to be contributory even when the cause of death was unrelated to any infection.

Overall, our national surveillance identified very few COVID-19 deaths in CYP irrespective of infection due to wild-type, Alpha, or Delta variant. IFR based on confirmed SARS-CoV-2 infections overestimates the risk because there was very limited testing for the virus until June 2020. Also, children with asymptomatic or mild, transient infection are less likely to be tested. We, therefore, used estimated infection rates using real-time modelling developed and updated regularly since the start of the pandemic to calculate age-specific and variant-specific IFR for CYP with confirmed SARS-CoV-2 infection. The estimated IFR of 0.7/100,000 was 4-fold lower than the 2.8/100,000 calculated using confirmed infections in England.

By using time intervals when the major variants were dominant in England, we also estimated lower IFR with the Delta variant compared to the Alpha variant or the wild-type strain. This is in contrast to adults, where the Delta variant was associated with more severe disease and death compared to previous variants [47]. Because of higher cases numbers, however, there were more deaths during the Delta variant wave compared to previous waves. The characteristics of CYP who died during the Delta wave were similar to those who died during the first year of the pandemic in England [15]. With the implementation of COVID-19 vaccination for adolescents and, more recently, for 5 to 11 year olds, it is hoped that those at increased risk will be protected against severe and fatal COVID-19.

### Strengths and limitations

The strength of this study is in the rapid initiation of enhanced national surveillance, including follow-up of deaths associated with SARS-CoV-2 infection, in CYP across England. Using multiple data sources to identify COVID-19 deaths with reasonable certainty, we have demonstrated the limitations of both time interval and death registration data for routine reporting of COVID-19 deaths in CYP, highlighting the critical importance of detailed case reviews in assessing rare outcomes in CYP infected with a highly prevalent virus.

There are some limitations. We did not have direct access to clinical case notes or records of perimortem events to conduct a thorough case review to ascertain the cause of death. However, we used our experience with national surveillance of vaccine-preventable diseases to rapidly follow-up fatal cases using multiple national data sources, clinical questionnaires, and establishing dialogue with relevant specialists, as required [19,20,48]. Reassuringly, our findings were very similar to the previous English study [15], which used a different methodology and had access to in-depth clinical data. The limited clinical data in our surveillance meant that we could not perform detailed analysis on underlying conditions in CYP who died of COVID-19. Additionally, the low numbers of COVID-19 deaths in CYP did not allow us to conduct subgroup analysis, such as trends by age over time, variant, or vaccination status. Also, some children may not have been included in our surveillance due to testing negative for SARS-CoV-2 at the time or not being tested prior to death, even though they may have had a prior infection, while others may have died subsequently as a result of an unknown or unidentified complication of prior COVID-19 infection. We also did not compare COVID-19 deaths with all-cause childhood deaths over the same period, as was done previously for the first year of the pandemic [11]. In that analysis, for example, while neurodisability was an important risk factor for severe and fatal COVID-19, SARS-CoV-2 was identified to contribute to only 3% of deaths in this vulnerable group. Additionally, we did not include PIMS-TS fatalities because this is a post-infectious immune-mediated condition and most cases are not positive for SARS-CoV-2 at diagnosis [49]. Finally, our surveillance period did not include the most recent pandemic wave due to the Omicron variant which is now the predominant variant globally.

## Conclusions

Ongoing national surveillance continues to demonstrate a very low risk of death in CYP with confirmed SARS-CoV-2 infection. Because of the high rates of asymptomatic and mild infections, deaths within 30 days of infection and death registrations both substantially overestimate fatalities in CYP, highlighting the critical importance of individual case reviews to assess such rare outcomes.

## Supporting information

S1 STROBE Statement Checklist of items that should be included in reports of observational studies. (DOCX)

## Abbreviations

CDC: Center for Disease Prevention and Control
CI: confidence interval
COVID-19: Coronavirus Disease 2019
CYP: children and young people
HES: Hospital Episodes Statistics
IFR: Infection fatality rate
IQR: interquartile range
LFD: lateral-flow device
MIS-C: multisystem inflammatory syndrome in children
NHS: National Health Service
ONS: Office for National Statistics
PDS: Personal Demographic Service
PIMS-TS: paediatric inflammatory multisystem syndrome
SARS-CoV-2: Severe Acute Respiratory Syndrome Coronavirus 2
SGSS: Second-Generation Surveillance System
UKHSA: UK Health Security Agency
